# Printable Gel Polymer Electrolytes for Solid-State Printed Supercapacitors

**DOI:** 10.3390/ma14020316

**Published:** 2021-01-09

**Authors:** Myeong-Lok Seol, Inho Nam, Ellie Sadatian, Nabanita Dutta, Jin-Woo Han, M. Meyyappan

**Affiliations:** 1 Center for Nanotechnology, NASA Ames Research Center, Moffett Field, CA 94035, USA; myeonglok.seol@nasa.gov (M.-L.S.); ssadatia@eng.ucsd.edu (E.S.); go2nabanita@gmail.com (N.D.); m.meyyappan@nasa.gov (M.M.); 2 Universities Space Research Association, NASA Ames Research Center, Moffett Field, CA 94035, USA; 3 School of Chemical Engineering and Materials Science, Department of Intelligent Energy and Industry, Institute of Energy Converting Soft Materials, Chung-Ang University, Seoul 06974, Korea; inhonam@cau.ac.kr

**Keywords:** solid-state supercapacitors, printing, gel polymer electrolyte, in-plane manufacturing

## Abstract

Supercapacitors prepared by printing allow a simple manufacturing process, easy customization, high material efficiency and wide substrate compatibility. While printable active layers have been widely studied, printable electrolytes have not been thoroughly investigated despite their importance. A printable electrolyte should not only have high ionic conductivity, but also proper viscosity, small particle size and chemical stability. Here, gel-polymer electrolytes (GPE) that are compatible with printing were developed and their electrochemical performance was analyzed. Five GPE formulations based on various polymer-conductive substance combinations were investigated. Among them, GPE made of polyvinylidene difluoride (PVDF) polymer matrix and LiClO_4_ conductive substance exhibited the best electrochemical performance, with a gravimetric capacitance of 176.4 F/g and areal capacitance of 152.7 mF/cm^2^ at a potential scan rate of 10 mV/s. The in-depth study of the in-plane solid-state supercapacitors based on various printed GPEs suggests that printable electrolytes provide desirable attributes for high-performance printed energy devices such as supercapacitors, batteries, fuel cells and dye-sensitized solar cells.

## 1. Introduction

Supercapacitors are energy storage devices based on the electrical double layer formation (electrical double layer capacitor, EDLC) or surface Faradaic reaction of nanomaterials (pseudocapacitor). They are attractive where fast and stable energy supply and uptake is needed due to their high power density, electrical durability and operation stability compared to batteries. Electrolyte is an essential supercapacitor component that creates the electrical double layer of an EDLC or activates the Faradaic reaction of a pseudocapacitor [1]. Depending on their physical states, electrolytes are classified into liquid electrolytes, solid electrolytes and gel-polymer electrolytes (GPEs). Liquid electrolytes provide high ionic conductivity but they are often flammable and require a confinement method such as hard packaging. Solid electrolytes ensure safe operation but they suffer from low ionic conductivity, limiting the energy storage performance. GPE possesses the best combination of the high ionic conductivity of a liquid electrolyte and good operation safety of a solid electrolyte [2].

Among various manufacturing techniques, printing, i.e., additive manufacturing, has attracted attention recently to produce a wide range of devices. A printed supercapacitor is fabricated by sequential layer-by-layer deposition of functional components with in-plane interdigitated structures made by precise patterning [3,4,5]. Printing has the benefits of low material waste, minimal fabrication facility, easy structural customization and good substrate compatibility, among others [6]. To develop a practical printed supercapacitor, it is important that all functional layers are printable, while minimizing the performance compromise from any process or material modification. While there have been numerous studies on printable active layers [7,8,9,10,11,12,13,14], the subject of developing and analyzing electrolytes for printed supercapacitors has received much less attention. Making printable electrolytes is challenging due to the difficulties in simultaneously achieving good electrochemical characteristics and proper physical characteristics needed for stable manufacturing. Ideally, printable electrolyte ink should have a proper viscosity, high firmness to maintain the printed structure, small particle size to prevent nozzle clogging and high ionic conductivity. Liquid electrolytes are not amenable for pattern confinement, whereas solid electrolytes can cause nozzle clogging owing to the coarse particles. GPEs, in contrast, have the suitable physical characteristics for printing; in addition, they can be easily configured to any desired size and shape, making them advantageous for modern in-plane manufacturing.

In this work, printable GPEs were developed and supercapacitors based on these printable GPEs were fabricated to analyze their electrochemical performance. Graphene-Mn_3_O_4_ nanocomposite-based pseudocapacitor electrodes were used for the device testing. All functional components, such as the current collector, electrodes and electrolytes, were fabricated by sequential printing steps. Here, the printing protocol for reliable fabrication is discussed first, followed by the introduction of electrolyte formulations for testing. Then, the electrochemical performance of the tested formulations is analyzed for comparison. Finally, the printed supercapacitor with the optimized electrolyte is analyzed in depth to evaluate its practical performance.

## 2. Materials and Methods

### 2.1. Materials

Polyvinyl alcohol (PVA), polyvinylidene difluoride (PVDF), polymethyl methacrylate (PMMA), phosphoric acid (H_3_PO_4_), sodium sulfate (Na_2_SO_4_), lithium perchlorate (LiClO_4_), ethyl cellulose, methanol, terpineol (95 wt.%), propylene carbonate (PC), and carbon nanotube (CNT) were purchased from Sigma Aldrich (St. Louis, MO, USA). Silver nanoparticle ink was purchased from Voltera (Kitchener, ON, Canada). Graphene nanoflake powder was obtained from CELTIG (Gatlinburg, TN, USA). Graphene-Mn_3_O_4_ nanocomposite (50 wt.% Mn_3_O_4_) was purchased from U.S Research Nanomaterials (Houston, TX, USA).

### 2.2. Gel Polymer Electrolytes Ink Synthesis

Five GPE formulations were used for the experiments: three of them were aqueous and the rest were based on organic solvents. For the aqueous GPEs, PVA was used because of its high solubility in water. Firstly, 5 g PVA was added to 40 mL deionized water in a beaker. The beaker was then inserted in a larger beaker filled with water, and was heated to 80 °C under magnetic stirring. The heated bath method prevents local overheating of PVA at the bottom of the beaker. A conductive substance (H_3_PO_4_, Na_2_SO_4_, or LiClO_4_) was gradually added to make a solution concentration of 0.5 M. After 30 min of heating, the solution was cooled to room temperature. The resulting aqueous GPE was then transferred to an empty printing tube for the manufacturing of the supercapacitor. For the organic GPEs, PC was used as the solvent. A total of 1.59 g LiClO_4_ as the ion-supporting salt was added in 30 mL PC to create 0.5 M solution, and PVDF or PMMA as the polymer matrix was added with a molar ratio of 3:1 (polymer:ion). The solution with conductive substances and polymer matrix was heated at 140 °C for 30 min under magnetic stirring. The resulting organic GPE was then transferred to an empty printing tube for the experiments.

### 2.3. Printed Supercapacitor Fabrication

The in-plane solid-state supercapacitors were fabricated by the sequential printing process based on the direct writing mechanism [3]. The devices are based on the pseudocapacitive mechanism using graphene-Mn_3_O_4_ nanocomposite electrodes [15,16]. Firstly, the current collector layer made of Ag nanoparticles was printed and annealed at 120 °C for 30 min. Then, the custom-synthesized active layer ink was printed on the current collector, followed by annealing at 100 °C for 10 min. The current collector and active layer have an interdigitated structure, enabling an in-plane and separator-free device. Morphological features are similar to the SEM and TEM images provided in our previous work [3]. Finally, the GPE inks were printed based on the four-step process to be explained in the next section. The printing parameters are as follows: probe pitch of 5.0 mm, pass spacing of 0.15 mm, feed rate of 500 mm min^−1^, trim length of 30 mm, trace penetration of 0.15 mm, anti-stringing distance of 2.0 mm, soft start ratio of 0.05 mm, soft stop ratio of 0.05 mm and rheological set point of 0.16.

### 2.4. Characterization

The electrochemical analyses including cyclic voltammetry (CV), galvanostatic charge and discharge (GCD) and electrochemical impedance spectroscopy (EIS) were conducted using a potentiostat (SP-150, Biologic). Both CV and GCD were measured for 10 cycles and the data from the last cycle are presented in the figures. The default potential scan rate for the CV measurements was 10 mV/s and voltage sweep range was from 0 to 0.8 V unless otherwise noted. For the EIS measurement, the voltage of the working electrode was 0.4 V and the frequency sweep range was from 0.1 Hz to 1 MHz. The areal performance metrics were calculated based on the active electrode area (60.7% of the total area).

## 3. Results and Discussion

### 3.1. Printing Protocols and GPE Formulations

**Figure 1:**
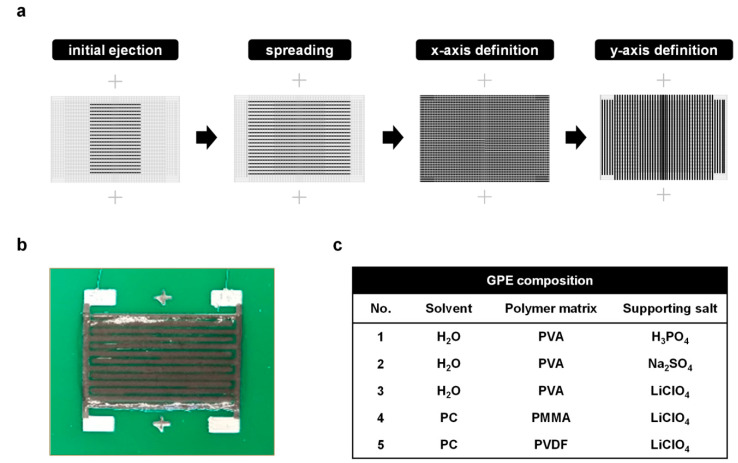
Fabrication process and results. (**a**) Pattern structures of the four-step electrolyte ink printing. The light gray area represents the entire pattern, while the black lines show the nozzle paths of the specific printing step. (**b**) Top-view image of the all-printed supercapacitor with the printed gel-polymer electrolyte (GPE). (**c**) Composition of the GPE formulations used in the experiments. Concentrations of the salts are 0.5 M and the molar ratio of polymer matrix and supporting salt is 3:1.

GPE printing is highly dependent on the ink viscosity. Low viscosity allows uniform thickness but is accompanied by overspreading. High viscosity ensures good pattern confinement but often leads to uneven surface and poor printing stability. In this study, a four-step printing process was optimized to create a uniform and consistent pattern (Figure 1a). The purpose of the first two steps is to supply a large amount of ink on the device, while the latter steps mainly help to distribute the ink to cover the entire device area. The fine dispenser employed here serves the purpose of ink supplying as well as physical spreading so that the electrolyte can cover the entire device area uniformly. In the first step, a significant amount of ink was supplied to the narrow center area. The second step covered a larger device area, but the amount of ink was less than in the first step. The purpose of this step is to distribute the ink in the x-direction and to supply additional ink to the edges. In the third step, the nozzle physically spread the existing ink in the x-direction, covering the entire electrode area. In the fourth step, the nozzles swept the ink in the y-direction, further improving the uniformity of the pattern. It is important to ensure that the electrolyte does not reach the contact pad as it can compromise the integrity of the device. After this step, a rectangular GPE pattern that covered the entire in-plane interdigitated structure of the electrodes was finalized (Figure 1b).

Five GPE ink formulations were selected and developed (Figure 1c). Formulations 1, 2, and 3 were aqueous electrolytes with the advantages of high ionic conductivity, non-flammability and a relatively simple manufacturing process. These formulations commonly used PVA as the polymer matrix, but employed different conductive substances: H_3_PO_4_, Na_2_SO_4_, and LiClO_4_, respectively. H_3_PO_4_ is widely used in aqueous electrolytes owing to its high ion mobility, but it can cause unwanted side reactions due to its high acidity. Na_2_SO_4_ is widely used as a neutral conductive substance because of its high stability and wide voltage window [17,18]. LiClO_4_ has recently gained industrial attention because of the high involvement of Li ions, stemming from the large size difference between the anion (ClO_4_^−^) and the cation (Li^+^). LiClO_4_ dissolves well in most polymer matrices because of the coordination interactions between the polar groups at the polymer hosts and Li ions [19,20]. Formulations 3, 4, and 5 were based on the same conductive substance (LiClO_4_) but different polymer matrices: PVA, PMMA, and PVDF, respectively. PVA has been widely used due to its high mechanical strength, non-toxicity and high chemical and thermal stability [21,22,23]. PMMA has good mechanical properties and compatibility to various salt materials [24,25,26]. PVDF also has good affinity and stability when combined with most salt materials [27,28]. The fluorine atoms in the PVDF backbone promote the dissociation of lithium salts into lithium ions, thereby increasing the ionic conductivity [28]. Room temperature ionic liquids may have viscosity comparable to some GPEs but they have not been considered here, as it is beyond the scope [29]. There are also deposition first-then-firming methods, but they were not considered because they require additional steps and instruments and additional labor [30].

### 3.2. Performance Comparison Between GPEs

**Figure 2:**
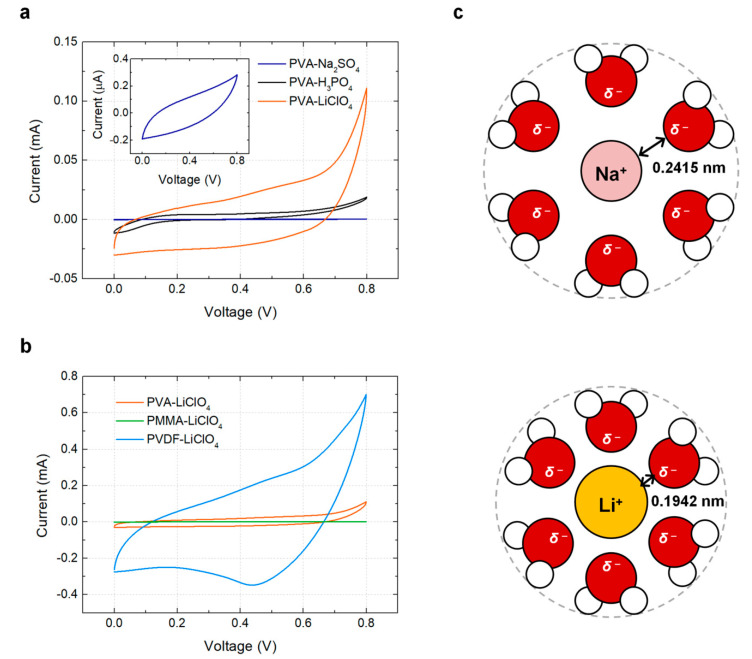
Performance comparison between GPEs. (**a**) Cyclic voltammetry results of the printed supercapacitors using aqueous GPEs with various supporting salts (Formulations 1, 2 and 3). The inset shows a magnified view of the PVA–Na_2_SO_4_ curve. (**b**) Cyclic voltammetry results for GPEs with various polymer matrices (Formulations 3, 4 and 5). (**c**) Schematic images of the hydrate forms of Na^+^ and Li^+^.

Figure 2a shows the CV curves of the supercapacitors for printed aqueous GPEs with various supporting salts (Formulations 1, 2 and 3) at a potential scan rate of 10 mV/s. The PVA–Na_2_SO_4_ GPE has a rectangular CV curve, proving the dominant capacitive behavior. The output value is relatively lower than those of the other two GPEs. Due to lower ionic conductivities, the equivalent series resistances of supercapacitors with neutral electrolytes are generally higher than those with acidic electrolytes [31,32,33]. The PVA–H_3_PO_4_ supercapacitor has a larger CV curve area. H_3_PO_4_ is known to promote the active Faradaic reaction of Mn_3_O_4_-based supercapacitors during charging and discharging. However, the PVA–H_3_PO_4_ supercapacitor has poorer stability than neutral electrolytes because of the excessively high reactivity and small potential window. As acidic electrolytes have a significantly higher H^+^ concentration than neutral electrolytes, there is a lower overpotential for hydrogen and oxygen evolution reaction, thereby decreasing the electrochemical stability and lowering the potential windows [34]. Therefore, LiClO_4_ was tested as an alternative neutral conductive substance. PVA–LiClO_4_ GPE presents better performance with a gravimetric capacitance of 2.33 F/g and an areal capacitance of 2.01 mF/cm^2^ compared to the other two aqueous electrolytes. The high performance is attributed to the excellent electrosorption property and fast surface redox reaction, which come from the significantly small size of hydrated lithium ions. A hydrated lithium ion (Li(H_2_O)^4+^) has an average Li–O distance of 0.1942 nm. Meanwhile, a hydrated sodium ion (Na(H_2_O)^6+^) has an average Na–O distance of 0.2415 nm [35]. Considering that the hydrate oxygen distance is 0.134 nm, the ionic radii of the hydrated four-coordinate lithium ion and six-coordinate sodium ion are 0.060 nm and 0.107 nm, respectively. The small size of the hydrated lithium ions results in the efficient adsorption/desorption or intercalation/deintercalation of electrolyte cations to the manganese oxide nanoparticles in the electrode, thereby changing the manganese oxidation state between III and IV during charging and discharging, respectively [36].

Although studies have mainly focused on aqueous GPEs, the current commercial market is dominated by organic GPEs, mostly owing to their high potential window. In addition, organic electrolytes allow wider material selection options for a current collector and packaging. Figure 2b presents the CV curves of printed supercapacitors with printed organic GPEs and various polymer matrices (Formulations 4 and 5) at a potential scan rate of 10 mV/s. For comparison, PVA–LiClO_4_–water data (Formulation 3) were also included in the plot. PMMA-based GPE presents the lowest capacity, while PVDF-based GPE shows the highest capacity, with a gravimetric capacitance of 23.15 F/g and an areal capacitance of 20.03 mF/cm^2^. While the salt and solvent solely determine the characteristics of the liquid electrolytes, the performance of the GPE is affected by the selection of polymers because polar groups in the polymer can significantly participate in lithium ion coordination and salt dissociation [37]. The interaction between the lithium ions and polymers influences the number of free charge species and ion conductivity. The poor performance of PMMA-based GPE can be attributed to its low ionic conductivity, which was compromised by the high viscosity. Bohnke et al. reported that the conductivity of a PMMA–LiClO_4_ membrane decreased considerably when the PMMA concentration was increased, owing to the interactions between the conducting electrolytes and polymer chains [24]. On the other hand, PVDF–LiClO_4_ GPE shows significantly higher electrochemical performance compared to the other formulations. An in-depth study of the PVDF–LiClO_4_ GPE is presented in the next section.

### 3.3. In-Depth Electrochemical Analysis

**Figure 3:**
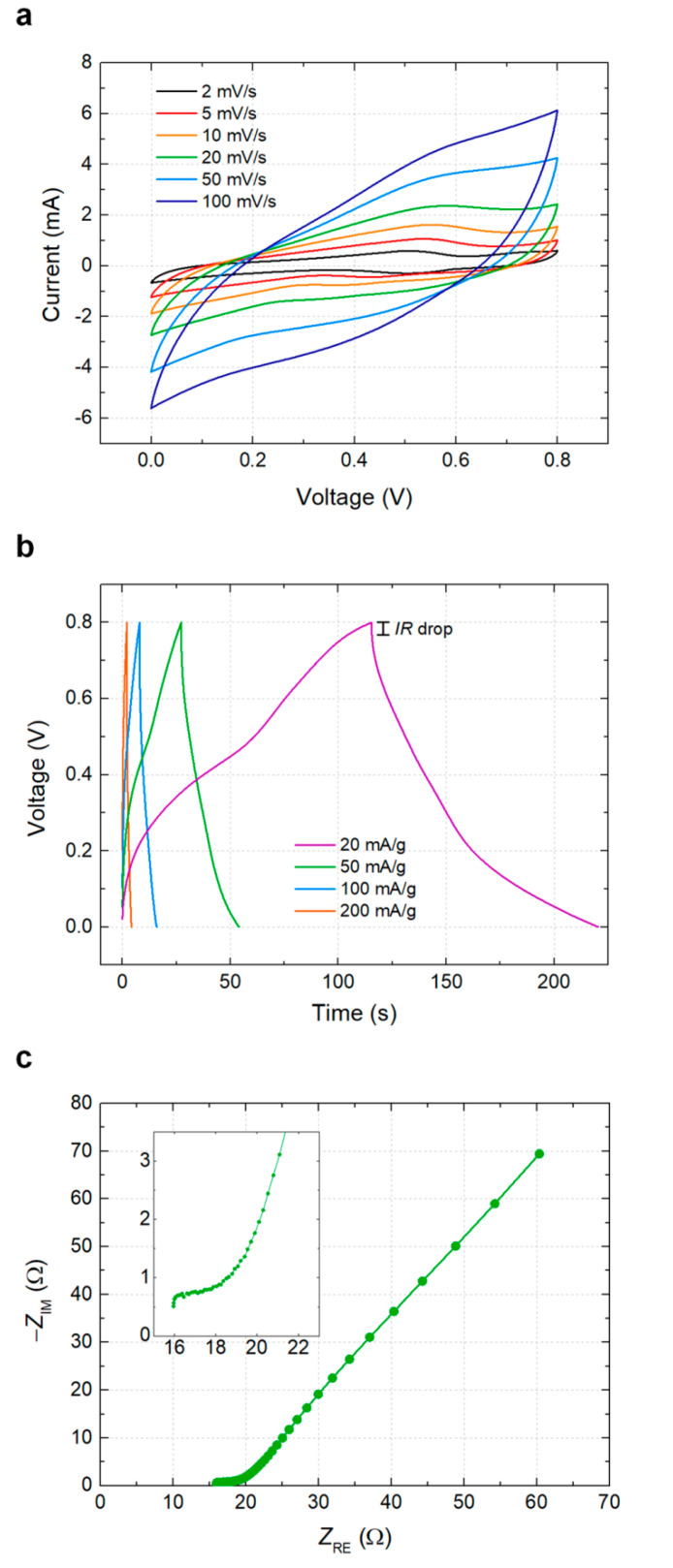
Electrochemical analysis of the printed supercapacitor with PVDF-LiClO_4_ GPE. (**a**) Cyclic voltammetry results at various scan rates. (**b**) Galvanostatic charge and discharge results at various current densities. (**c**) Electrochemical impedance spectroscopy results.

The electrochemical performance of the printed solid-state supercapacitor with PVDF–LiClO_4_–PC GPE was analyzed at various scan rates (Figure 3). The CV scans exhibit a semi-rectangular shape at different scan rates (Figure 3a), which shows that the capacitive behavior of the device is based on the fast ion transport. The devices feature a triangular galvanostatic charge/discharge curve at various current densities (20–200 mA/g) (Figure 3b), implying the ideal charge balance and capacitive behavior between the symmetric electrodes [24]. The Nyquist plot shows an equivalent series resistance of 16 Ω. The diameter of the semicircle decreased with increasing frequency, proving that the interface resistance between the electrode and the electrolyte is sufficiently low (Figure 3c). It is well known that lithium ions are efficiently transferred along oxygen atoms in amorphous polymer segments [38]. In the PVDF–LiClO_4_–PC system, the oxygen atoms in the PVDF bridge different lithium ions and coordinate to them. Lithium ions hop from one coordination site to another through ligand exchange in the GPE structure [39]. Conductivity based on the lithium ion hopping mechanism through ligand exchange occurs in highly packed electrolytes [40], and PVDF provides a tightly packed coordination. Therefore, the interactions between the lithium ions as Lewis acid and the abundant hopping sites of oxygen atoms in PVDF result in the high conductivity of the printed supercapacitor.

**Figure 4:**
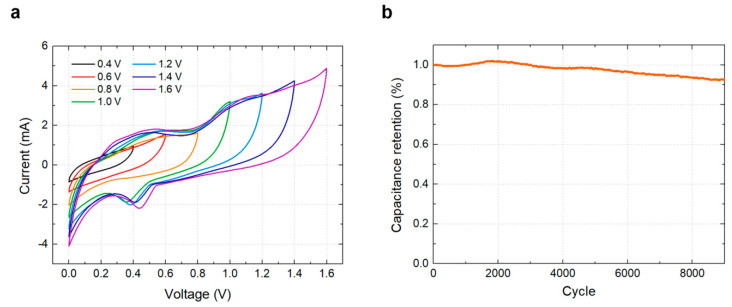
Voltage dependence and cyclic durability of the printed supercapacitor with PVDF-LiClO_4_ GPE. (**a**) Cyclic voltammetry results at various voltage scanning ranges. (**b**) Cyclic endurance test result with 9000 charge/discharge cycles.

The cell operation voltage is a crucial factor in determining the energy and power densities. To confirm the voltage window of the optimized printable GPE, CV characteristics were measured at the scanning voltage ranging from 0.4 V to 1.6 V with a step voltage shift of 0.2 V (Figure 4a). The result confirms that the electrolyte is stable at the voltage higher than the water oxidation potential (i.e., oxygen evolution potential). Although the voltage range was not pushed to the limit here due to the concern of the possible presence of water in the ambient, a higher voltage range will be available considering the nature of organic electrolytes. At a scanning voltage of 1.6 V, the printed supercapacitor provided a gravimetric capacitance of 176.4 F/g and areal capacitance of 152.7 mF/cm^2^, and good cyclic durability over 9000 charge/discharge cycles with negligible performance degradation (Figure 4b). Any degradation is mainly caused by the interaction between the electrolyte and air, and can be mitigated by forming a passivation layer [41]. These results demonstrate that the optimized printed GPE employed in this work satisfies the standard performance requirements of practical energy storage applications.

## 4. Conclusions

In this work, printable electrolytes were developed for in-plane solid-state printed supercapacitors and their electrochemical performance was analyzed. The four-step sequential printing technique employed here allowed stable printability as well as good pattern confinement for the printed GPE layers. Among the formulations evaluated, the combination of PVDF as the polymer matrix and LiClO_4_ as the conductive substance exhibited the best performance. The PVDF–LiClO_4_–PC GPE provided high electrochemical performance, high durability against electrochemical stresses and excellent printing stability. The printable electrolytes enable end-to-end additive manufacturing of supercapacitors with minimal equipment requirements, maximized resource efficiency and efficient integration with other printed electronic components. In addition, other energy devices that require an electrolyte as an essential component such as batteries, fuel cells and dye-sensitized solar cells can also employ printable electrolytes in order to meet demands of high performance, stability and ease of printing needed in applications.

## Data Availability

The data presented in this study is contained within the article.
